# Long head of biceps tendon augmentation in rotator cuff repair enhances tendon healing, shoulder function and patient‐reported outcomes one‐year post‐surgery

**DOI:** 10.1002/jeo2.70033

**Published:** 2024-10-10

**Authors:** Pablo Cañete San Pastor, Inmaculada Prosper Ramos, Alberto Garcia Roig, Joan Andreu Safont

**Affiliations:** ^1^ Doctoral School, Catholic Unversity of Valencia San Vicente Martir Hospital de Manises Manises Valencia Spain; ^2^ Hospital de Manises Valencia Spain

**Keywords:** cuff re‐rupture, long head of the biceps, rotator cuff repair, superior capsular reconstruction

## Abstract

**Purpose:**

The aim is to determine the effect on healing and functionality of patients after 1 year of biceps augmentation of a rotator cuff repair (RCR) compared to RCR plus long head of the biceps (LHB) tenotomy. In addition, to analyse the main factors involved in the recovery after the surgery.

**Methods:**

A prospective, comparative, non‐randomized study (Level of Evidence III) was conducted. Patients with repairable rotator cuff tears were allocated to either the control group, with a double row transosseous equivalent RCR with LHB tenotomy, or the RCR+augmentation with LHB group. Patients were evaluated for radiological (MRI), clinical (cuff size, Patte and Goutallier scales) and functional variables (Constant and American Shoulder and Elbow Surgeons [ASES] scales) before the intervention. At 1‐year follow‐up cuff healing was confirmed through MRI and functional evaluation with Constant, ASES, simple shoulder test [SST] and Disabilities of the Arm, Shoulder and Hand scales.

**Results:**

Seventy‐seven patients underwent control or RCR+augmentation with LHB, there were no preoperative differences between the groups. After 1 year of the surgery, re‐rupture occurred in 38.5% and 16% of the patients in control and RCR+augmentation with LHB groups, respectively (*p* = .026). Total functionality was higher (*p* < .05) in RCR+augmentation with LHB than in the control group: Constant, SST and ASES scales. Among the explored factors involved in healing, re‐rupture occurred in 100% of the cases with high fatty degeneration. Besides, higher initial functionality (Constant scale) and RCR+augmentation with LHB increased the odds of healing (odds ratio [OR] = 1.12 [1.04–1.21]; OR = 5 [1, 61], respectively), while higher cuff length had a detrimental effect (OR = 0.92 [0.85–0.99]).

**Conclusion:**

RCR+augmentation with LHB achieves a higher healing percentage and a better functional evolution than RCR+LHB tenotomy, 1 year after cuff repair. Fatty degeneration, cuff length and initial functionality are the main factors involved in cuff healing.

**Level of Evidence:**

Level III randomized controlled trial.

**Abbreviations**
ASES: American Shoulder and Elbow Surgeons Score
DASH: Disabilities of the Arm, Shoulder and Hand
IQR: interquartile range
LHB: long head of the biceps
MCID: minimal clinically important difference changes
MRI: magnetic resonance imaging
OR: odds ratio
RCR: rotator cuff repair
RoM: range of motion
SCR: superior capsular reconstruction
SD: standard deviation
SST: simple shoulder test
UCLA: University of California, Los Angeles
VAS: Visual Analog Scale

## INTRODUCTION

Rotator cuff repair (RCR) surgeries have increased exponentially in last years, together with the development of technical improvements in anchors, sutures, tapes and procedures to achieve a better recovery after RCR. But despite all these advances, rotator cuff re‐rupture is still being a common complication that can account for about 11%–94% according to the literature [22].

In a meta‐analysis that included more than 2600 RCRs, it was found that patients who presented rotator cuff retear showed worse clinical outcomes and lower strength compared to patients with a healed cuff [40].

One of the causes for so much variation in cuff retear could be the interaction of multiple biological and biomechanical factors that affect the rate of healing after RCR. As such, age, diabetes, smoking, vascular insufficiency, obesity, osteoporosis, hyperlipidemia, lifestyle, type of work, fatty infiltration of the rotator cuff muscles, size of the tear and type of the repair (single or double row) [1, 2, 3, 10, 19, 21, 24, 25, 26, 27, 31, 33].

Mihata et al. [25] described a procedure for superior capsular reconstruction (SCR) of the shoulder in the treatment of irreparable supraspinatus tears to prevent upper migration of the humeral head in order to regain function. This technique consists in the fixation of a fascia lata autograft in the upper part of the glenoid and greater tuberosity of the humerus. This procedure resulted in clinical improvement of the patients. The present study is based on this procedure, but focused on a kind of SCR with long head of the biceps (LHB), a biological augmentation with the LHB for the RCR in patients with repairable cuff tears. Possible improvements of this modification have already been suggested by Kim and colleagues, but in comparison with dermal patches [18]. SCR has already been performed with the LHB, with good mechanical and clinical results [13, 17, 34].

The objective of the present study was to determine the effect of RCR with LHB augmentation on healing and functionality after 1 year, compared to RCR with LHB tenotomy. The hypothesis is that LHB augmentation in a repairable rotator cuff increases tendon healing rates and improves patient clinical outcomes. Additionally, the study aimed to analyse the main factors involved in recovery after surgery.

## METHODS

### Study design

This prospective, comparative, non‐randomized study investigated the effect of augmenting RCR with the proximal stump of the long head of the biceps tendon (LHBT) compared to standard cuff repair. Eighty‐five patients with repairable rotator cuff tears underwent surgery by the same clinician (P.C.S.P.) between February 2021 and March 2022. Patients were sequentially assigned to one of two groups: the control group received a tension‐free double‐row equivalent transosseous repair and LHB tenotomy, while the experimental group received a tension‐free double‐row equivalent transosseous repair with augmentation using LHBT. Therefore, it was a controlled clinical trial with a level of evidence III.

### Sample size calculation

The minimum sample size was calculated in advance using the Biomath online tool [35], focusing on functionality as the main outcome measured by the Constant score. Previous studies indicated the following parameters for comparison after 1‐year follow‐up [4]: ☐1 = 81; ☐2 = 63; *n*1 = 56; *n*2 = 82; Sp = 9.9. Considering a significance level of 5% and a power of 80%, the minimum sample size for each group was nine participants. Given the meta‐analysis for augmentation with LHB techniques [38, 39] showing sample sizes ranging from 14 to 88 with significant differences for *n* = 20, the sample size for the present study was set at 20 participants per group to ensure robustness.

### Inclusion and exclusion criteria

Inclusion criteria were age over 18 years, symptomatic rotator cuff tears unresponsive to adequate rehabilitation, feasibility of a complete tension‐free double‐row repair, and a minimum follow‐up of 12 months. Exclusion criteria included massive irreparable tears, superior migration of the humeral head in Hamada phase 2 or higher [16], glenohumeral osteoarthritis, previous severe cervical disorders diagnosed via MRI (e.g., cervical nerve injuries, cervical arthrosis), axillary nerve paralysis, absence of LHB and partial RCR.

### Baseline and follow‐up evaluations

Patients were evaluated for radiological, clinical and functional variables before surgery. Functional scales used included the Constant score (assessing range of motion, strength, balance and pain) [21], the American Shoulder and Elbow Surgeons (ASES) score [38] and the Visual Analog Scale for pain (VAS) [20]. Tendon tear type was evaluated preoperatively using MRI. One year post‐surgery, tendon integrity was assessed via MRI, and functionality was measured using the Constant score, ASES, VAS, Disabilities of the Arm, Shoulder and Hand (DASH) score, and the simple shoulder test (SST) [23].

### Radiological assessment

**Figure 1:**
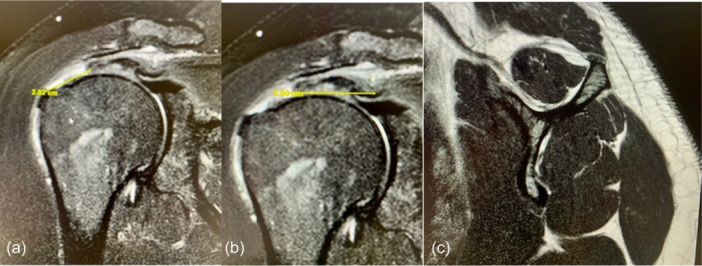
(a–c) Supraspinatus tear in a 64‐year‐old male. (a) Grade 2 in Patte classification with retraction to the humeral head; (b) long tendon stump of 35 mm; and (c) fatty degeneration Goutallier grade 2.

**Figure 2:**
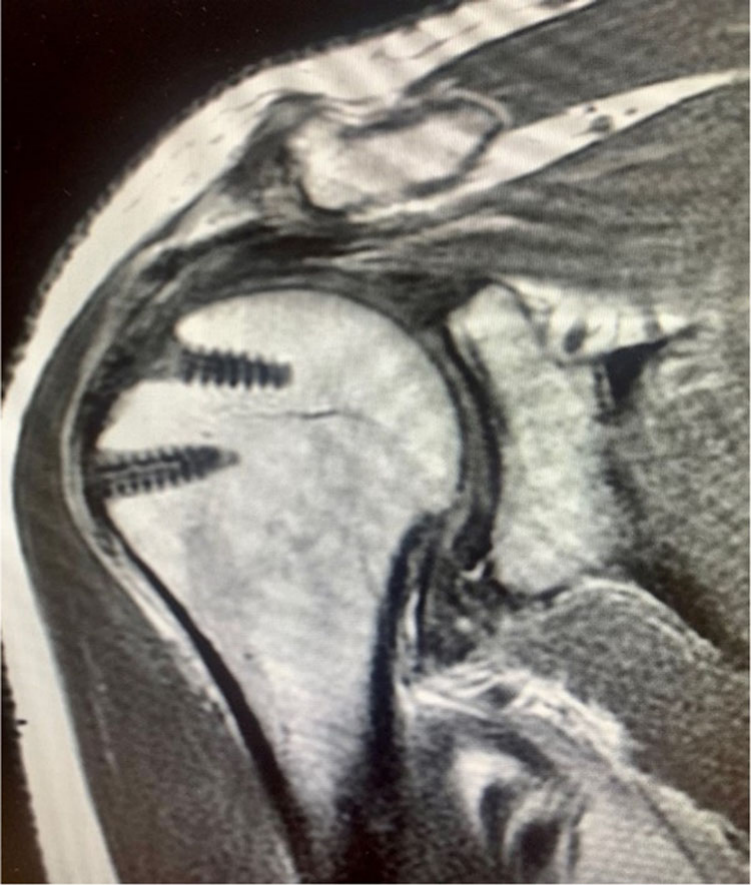
Correct healing of the tendon 1 year after the surgery, type 2 in the Sugaya classification.

Preoperative and 1‐year postoperative assessments were performed by a team of three radiologists experienced in shoulder pathology, blinded to patient allocation. Preoperative MRI assessed the number of affected tendons, tear size (Cofield classification) [8], rupture retraction (Patte classification) [30], tendon stump size and muscle fatty degeneration (Goutallier classification) [14]. Grades 3 and 4 indicated high fatty degeneration (Figure 1a–c). One‐year MRI evaluated RCR integrity using Sugaya's classification [28, 36]. The integrity criterion in this study was the one suggested by Yang et al. [40] to classify patients into two categories: healed and retear according to Sugaya's fourth and fifth grades like retear tendon, while Sugaya's grades 1, 2 and 3 were defined as a healed cuff (Figure 2).

### Surgical technique

**Figure 3:**
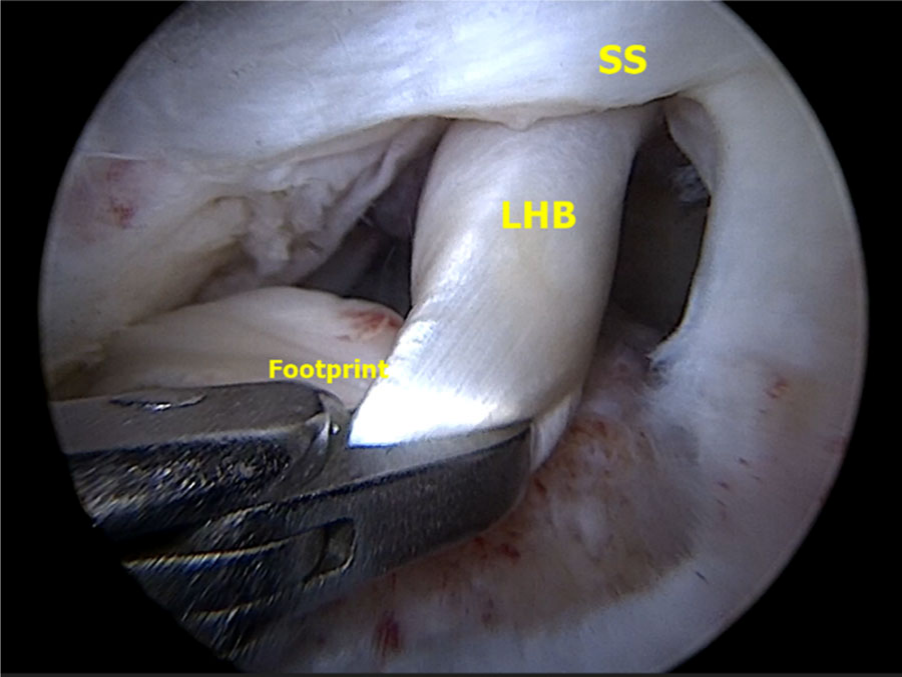
Shoulder arthroscopy of a right shoulder in lateral decubitus position. Subacromial view from a posterolateral portal. The mobility and integrity of the LHB should be checked with a tendon clamp. The LHB is subsequently repositioned to the middle of the greater tuberosity of the humeral head to ensure that it reaches correctly, before fixing. LHB, long head of the biceps; SS supraspinatus.

**Figure 4:**
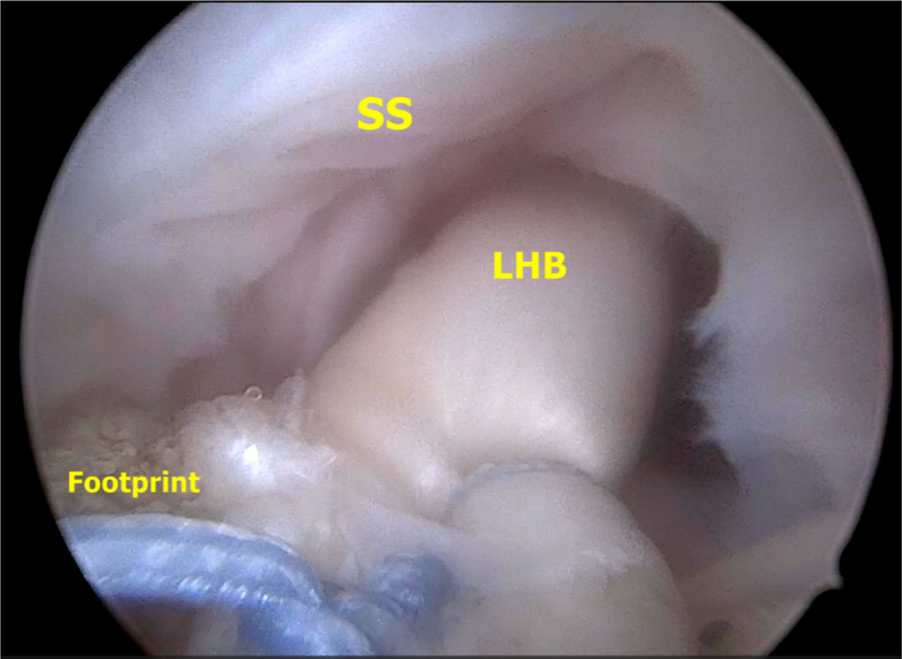
Shoulder arthroscopy of the right shoulder in lateral decubitus position. Subacromial view from a posterolateral portal. The LHB can be seen inserted in the glenoid and in the middle of the greater tuberosity of the humerus, functioning as the superior capsule, to prevent superior migration of the humeral head. We check the correct insertion and tension of the LHB. We can see that we have enough space to suture the supraspinatus and bring it to the footprint. LHB, long head of the biceps; SS, supraspinatus.

**Figure 5:**
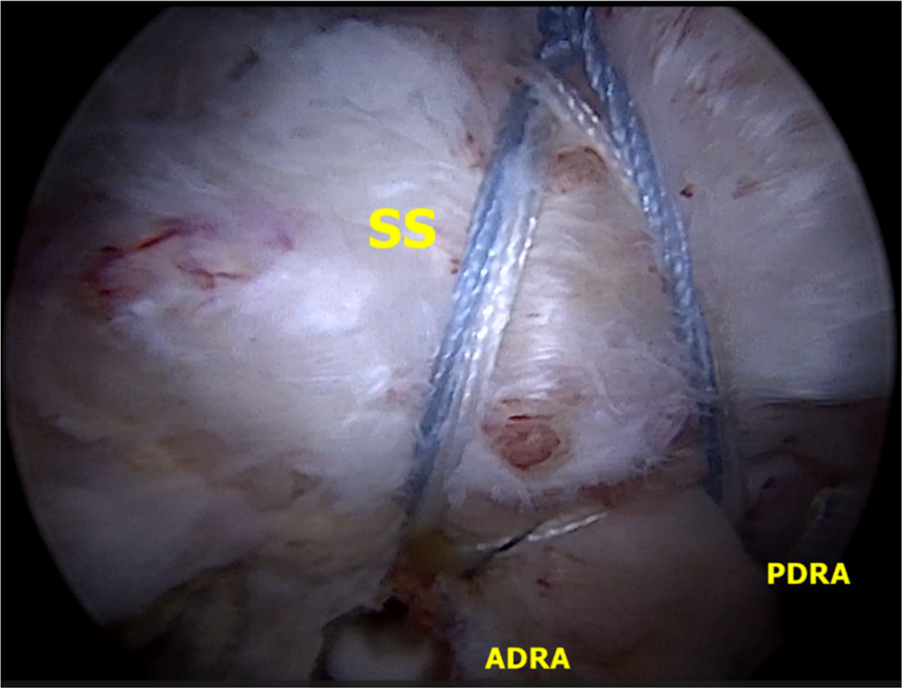
Shoulder arthroscopy of the right shoulder in lateral decubitus position. Subacromial view from lateral portal. Mattress sutures have been tied creating the medial.

All cuff repairs were performed arthroscopically with a tension‐free double‐row equivalent transosseous suture technique by the same surgeon, following Cañete et al.'s procedure [7, 29]. Key steps included preparing the greater tuberosity for tendon‐to‐bone healing, releasing the LHBT, ensuring its reach to the humeral head (Figure 3) and fixing it with a triple‐loaded anchor (5.5 mm, Biocomposite Bio‐corkscrew FT suture anchor, Arthrex). The LHBT was repositioned and fixed at the centre of the greater tuberosity (Figure 4), acting as augmentation. The supraspinatus tendon was then repaired using remaining sutures from the triple‐loaded anchor and additional anchors (5.5 mm Biocomposite Bio‐swivelock C, Arthrex), achieving a tension‐free double‐row repair (Figure 5).

### Ethical considerations

All data collection procedures were conducted after obtaining informed consent in accordance with the Declaration of Helsinki and with approval from the Ethics Committee of Hospital Universitario y Politécnico La Fe in Valencia (Spain).

### Statistical analysis

Statistical analysis was performed using IBM SPSS v24. Qualitative variables were described in absolute (*n*) and relative (%) frequencies, and relationships were analysed using Pearson's chi‐square test or Fisher's exact test. Quantitative variables were described as mean (*M*) with standard deviation (SD) and median (Md) with interquartile range (IQR), with normal distribution verified using the Kolmogorov–Smirnov test. Group means were compared using the *t*‐test for independent samples or Mann–Whitney *U* test, as appropriate. Within‐group comparisons used the paired *t*‐test or Wilcoxon test for normal or non‐normal variables, respectively.

Minimal clinically important difference (MCID) was calculated using baseline functional measurements (constant total score) of the two groups [11, 13], with pooled SD (Sp) × 0.5. Logistic regression analysis explored factors affecting cuff healing at 1‐year follow‐up, with healing as the outcome variable and age, experimental group, and initial clinical and functional characteristics as independent variables. Odds ratios with 95% confidence intervals were calculated. Variables with blank categories were excluded from the analysis. The significance level in all cases was *α* = .05.

The sample was composed for 85 patients who met selection criteria. Although according to the sample size calculation, 20 cases per group was enough to assess the difference between groups, we increased the size almost the double to get stronger results.

## RESULTS

**Figure 6:**
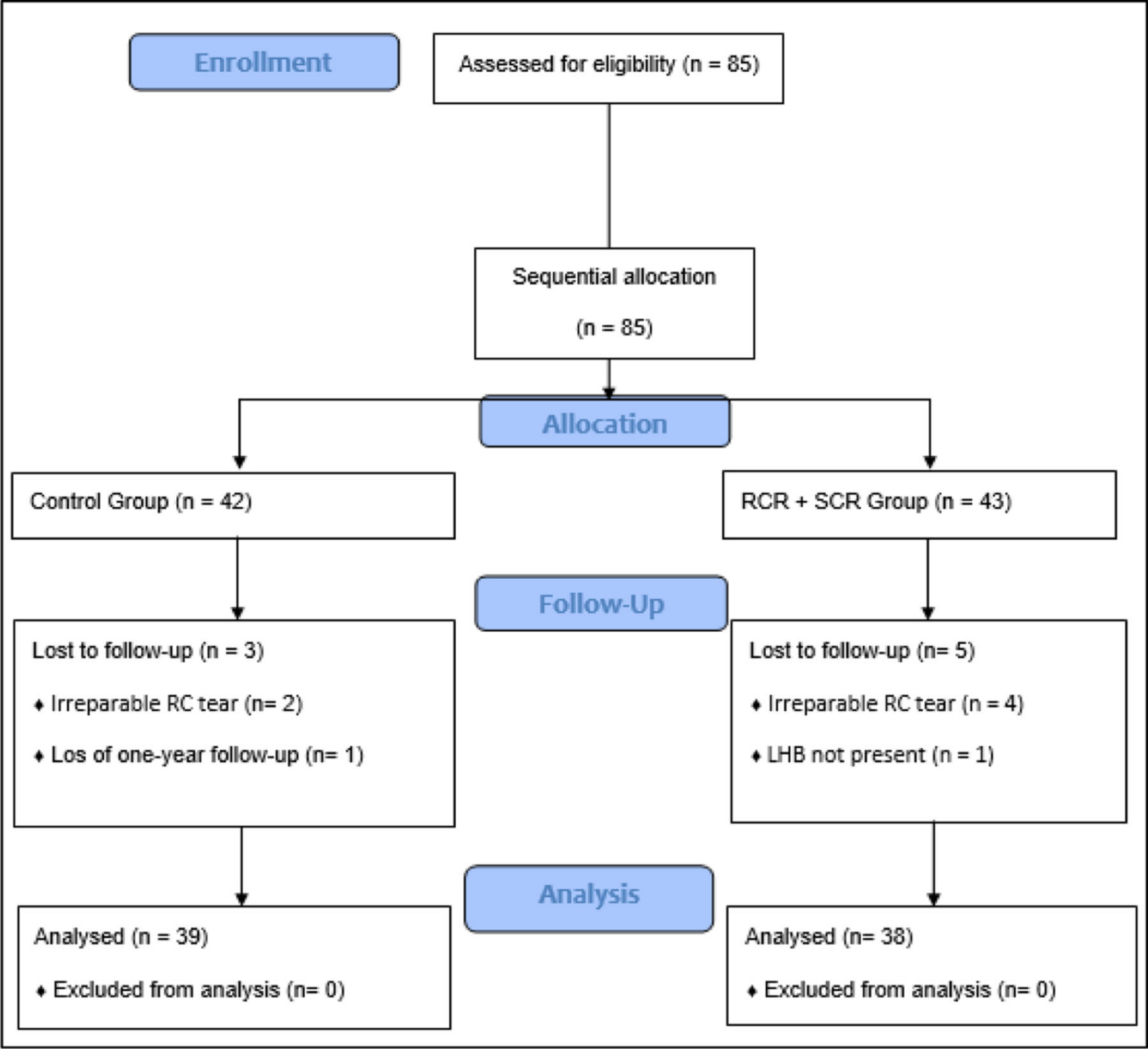
CONSORT diagram flow for enrolment, allocation and follow‐up of the sample. LHB, long head of the biceps; RC, rotator cuff; RCR, rotator cuff repair; SCR, superior capsular reconstruction (augmentation with the long head of the biceps tendon).

The allocation resulted in 42 and 43 patients for control and RCR+augmentation with LHB groups, respectively. The 1‐year follow‐up was possible only in 39 and 38 patients from each group. The CONSORT diagram flow chart is shown in Figure 6.

### Preoperative characteristics of the patients

Most of the patients, 63%, had a medium sagittal size (17 mm on average) according to the Cofield classification, and more than 50% were classified like Patte grade 2, with an average retraction of 19.27 mm. The muscular fatty degeneration in the affected shoulder was 1.63 points on the Goutallier scale with the majority of patients with grade 2. The average size of the tendon stump was 29 mm. The details of the descriptions can be seen in Table 1.

**Table 1:** Descriptive statistics of the experimental groups before the surgery.

	Control (*n* = 39)		RCR + SCR (*n* = 38)		
	*n* (%)		*n* (%)		*p* Value
Sex (m)	20 (51.3)		23 (60.5)		.414
Shoulder side (right)	25 (64.1)		24 (64.9)		.945
Tendons^a^ (2)	2 (5.1)		2 (5.3)		>.999

	***M*** ± **SD**	**Md [RIC]**	***M*** ± **SD**	**Md [IQR]**	***p* Value**
Age	60.66 ± 7,6	60 [12]	60.26 ± 7.83	61 [13]	.690
Retraction (mm)	19.05 ± 8.42	20 [15]	19.79 ± 9.9	20 [14]	.721
Sagital size (mm)	15.71 ± 6.78	15 [12]	18.34 ± 9.41	19 [11]	.278
Stump size (mm)	27.87 ± 8.52	30 [15]	30.18 ± 10.82	30 [18]	.597
Goutallier classification	1.63 ± 0.82	2 [1]	1.61 ± 0.68	2 [1]	.678
Constant scale (s)					
Pain	3.72 ± 2.49	5 [5]	3.82 ± 2,45	5 [5]	.858
Activity	9.03 ± 2.65	8 [2]	8.58 ± 2.02	8 [2]	.466
RoM	33.95 ± 5.53	36 [8]	34 ± 4.72	35 [7]	.765
Strength	6.56 ± 6.08	5 [10]	6.71 ± 4.76	5 [5]	.575
Total	52.87 ± 10.33	54 [17]	53.08 ± 6.84	53 [10]	.907
VAS (s)	8 ± 1.28	8 [2]	8 ± 1.32	8 [3]	.864
Escala ASES (s)					
Subtotal VD	14.08 ± 2.36	14 [3]	14.03 ± 2.28	14 [2]	.996
Total	33.46 ± 9.13	35 [12]	33.38 ± 9.11	36 [17]	.927

Abbreviations: ASES, American Shoulder and Elbow Surgeons; average, mean; IQR, interquartile range score; m, men in the group; Md, median; ROM, range of motion subscale; SD, standard deviation; VAS, Visual Analog Scale.

^a^ Number of affected tendons.

### Healing of patients at 1 year of follow‐up

**Figure 7:**
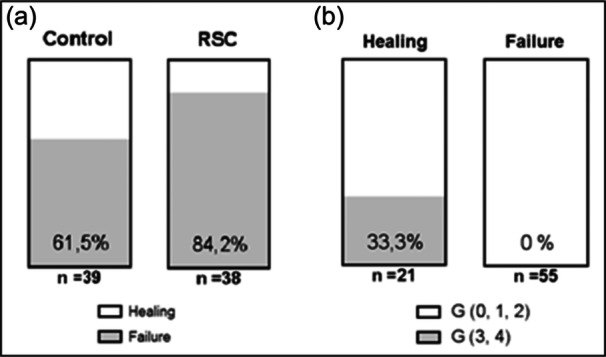
Effect of the intervention on healing 1 year after surgery. (a) Proportion of patients with correct healing in the experimental groups (*p* = .026). (b) Proportion of patients with healing according to the levels of the Goutallier scale (*p* < .001). G: reclassification of the Goutallier scale to two categories: levels 0, 1 and 2; levels 3 and 4.

One year after the surgery, a total of 56 patients, representing 72.7%, were found to have scores 1, 2 and 3 on the Sugaya scale, which was considered an adequate healing with tendon integrity (Figure 7). The group that received the surgery with RCR+augmentation with LHB had a higher percentage (84%) of patients with tendon integrity, compared to the control group, which presented a 61% of healed tendons thus RCR+augmentation with LHB had a statistically significant effect on healing at one‐year follow‐up (Table 2; *p* = .026).

**Table 2:** Relationship of tendon healing 1 year after the surgery, with the augmentation with LHB technique and initial factors.

	Re‐rupture (*n* = 21) *n* (%)	Healed cuff (*n* = 56) *n* (%)	*p* Value
Group			
Control	15 (38.5)	24 (61.5)	**.026**
RCR+LHB A	6 (15.8)	32 (84.2)	
Sex			
Women	10 (29.4)	24 (70.6)	.708
Men	11 (25.6)	32 (74.4)	
Shoulder			
Left	7 (25.9)	20 (74.1)	.845
Right	14 (28)	36 (72)	
Cofield			
Small	4 (16.7)	20 (83.3)	.134
Medium	14 (29.2)	34 (70.8)	
Large	3 (60)	2 (40)	
Stump size			
≤30 mm	15 (31.3)	33 (68.8)	.313
>30 mm	6 (20.7)	23 (79.3)	
Goutallier (2c)			
0, 1 y 2	14 (20.3)	56 (79.7)	**<.001**
3 y 4	7 (100)	0 (0)	

*Note*: Bold values indicate statistically significant at *p* < .05.

Abbreviations: 2c, 2 categories Goutallier classification; Cofield, sagital size classification; LHB A, long head biceps augmentation; RCR, rotator cuff repair.

**Figure 8:**
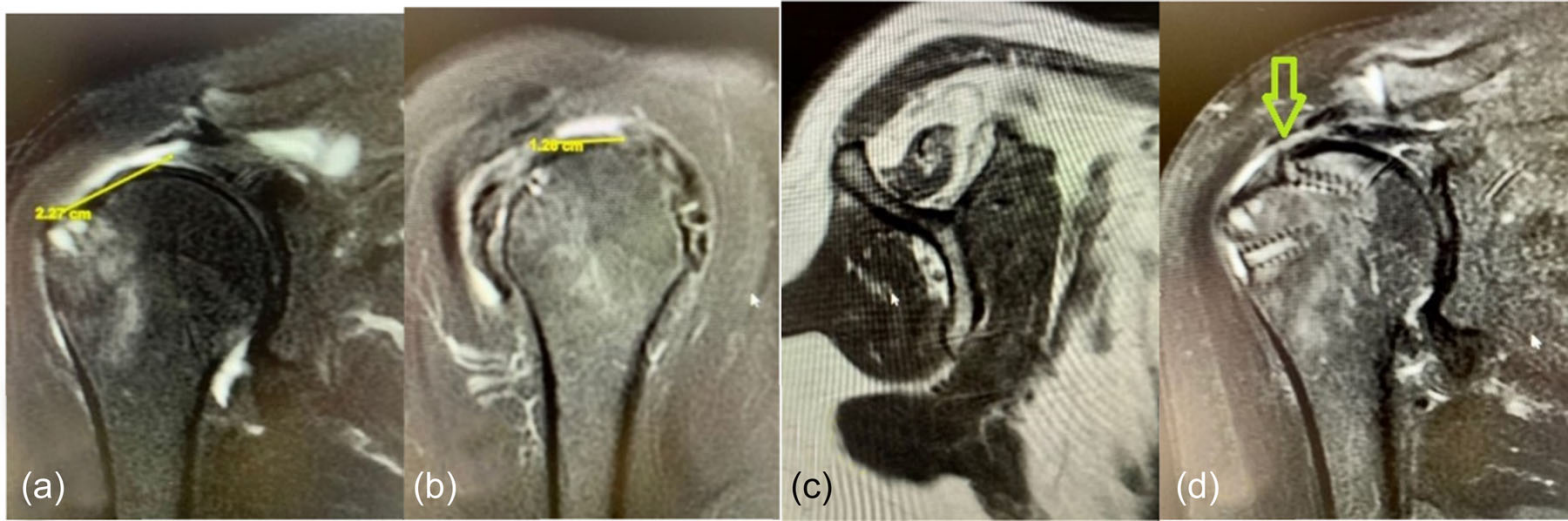
(a–d) Complete tear of the supraspinatus in a 67‐year‐old woman. (a) Grade 2 in Patte's classification with the tendon in the middle of the humeral head. (b) Medium size in the Cofield classification. (c) Grade 3 in the Goutallier classification, with a higher percentage of fat than muscle tissue. (d) Re‐rupture of the cuff, Sugaya grade 5, 1 year after the surgery (green arrow).

Besides, the proportions of sex, operated side, sagittal size (Cofield scale) and stump size were also compared, depending on whether it was greater or less than 30 mm, without finding an association of any of these factors with healing (*p* > .05). However, fatty degeneration according to the Goutallier scale in two categories did show a significant relationship with healing (*p* < .001), since none of the patients in the worst condition (grades 3 and 4) presented healing of the cuff (Figure 8a–d).

### Effect of RCR+augmentation with LHB on functionality after surgery

**Figure 9:**
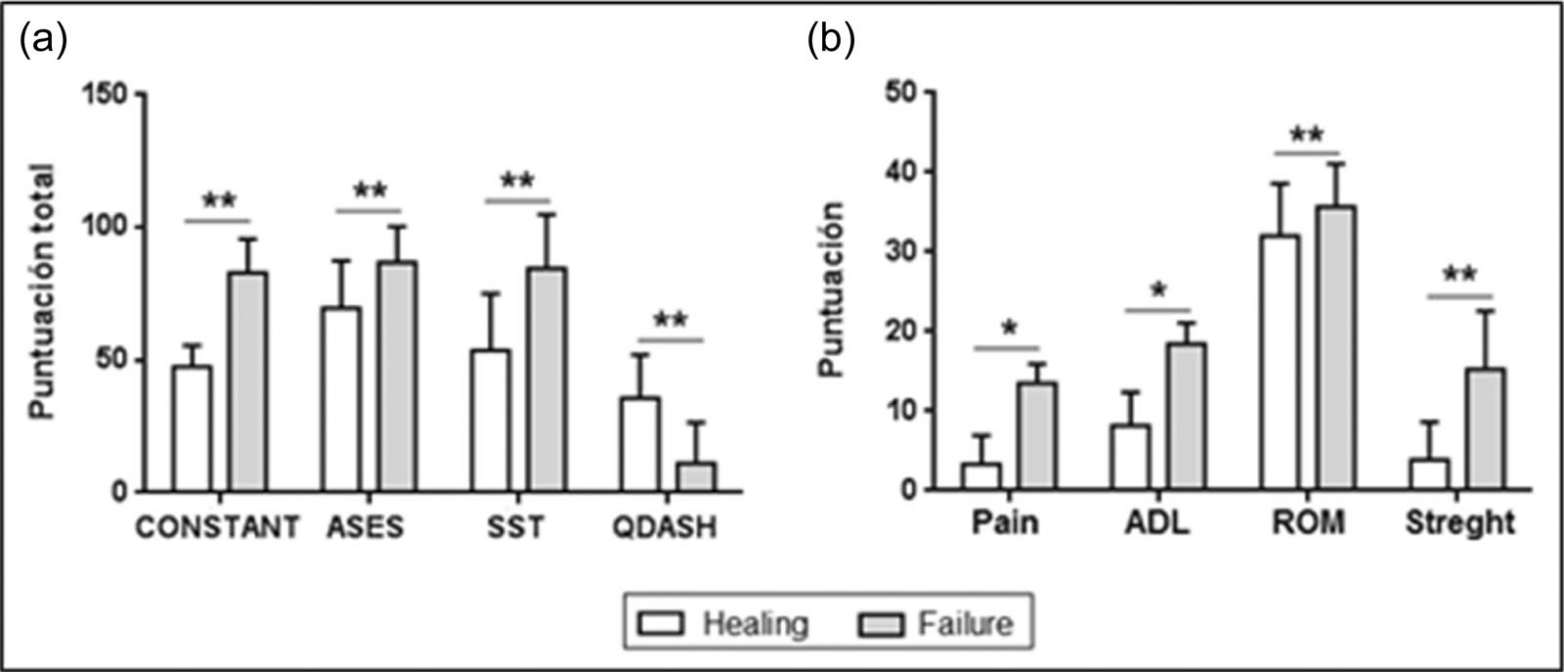
Functionality in the operated shoulder 1 year after surgery, according to the healing achieved. (a) Total scores. (b) Subscales of the Constant scale. *p < .05; **p < .01.

One year after surgery, patients who underwent RCR with LHB augmentation showed significantly better outcomes across all functional scales compared to the control group. They had higher total Constant scores (*p* = .007) and ASES scores (*p* = .029), with an approximate 10‐point difference on both scales (Table 3). In the SST, these patients scored 15 points higher than those in the control group (*p* = .014). For the QuickDASH, the LHB augmentation group had almost half the disability score of the control group (*p* = .038). Additionally, the Sugaya classification scores were lower in the RCR with the LHB augmentation group (*p* = .02). Figures 7 and 9 illustrate these results.

**Table 3:** Healing and functionality in control and augmentation with LHB patients 1 year after surgery.

	Control		RCR + SCR		
	*M* ± SD	Md [IQR]	*M *± SD	Md [IQR]	*p* Value
Sugaya	3.24 ± 1.15	3 [1]	2.58 ± 1.06	3 [1]	**.020**
Constant scale (s)					
Pain	12.61 ± 3.26	15 [4]	13.41 ± 2.53	15 [3]	.525
Activity	17.66 ± 3.71	20 [5]	18.11 ± 2.66	20 [5]	.981
RoM	31.68 ± 7.19	35 [9]	36.95 ± 3.06	38 [5]	**<.001**
Strength	11.4 ± 7.77	10 [15]	14.41 ± 7.63	14 [12]	.082
Total	73.34 ± 17.35	76 [25]	82.85 ± 10.91	84 [14]	**.007**
VAS (s)	1.84 ± 2.1	1 [4]	0.87 ± 1.42	0 [1]	.109
ASES (s)					
Subtotal VD	21.85 ± 6.55	23 [11]	24.74 ± 4.92	26 [6]	.053
Total	77.44 ± 18.46	83 [30]	86.89 ± 13.18	91 [10]	**.029**
SST (s)	68.26 ± 26.53	71 [42]	83,33 ± 20,5	83 [17]	**.014**
Q‐DASH (s)	22.79 ± 20.21	19 [28]	13.34 ± 16.87	7 [12]	**.038**

*Note*: Bold values indicate statistically significant at *p* < .05.

Abbreviations: ASES, American Shoulder and Elbow Surgeons; IQR, interquartile range; *M*, average; Md, median; Q‐DASH, Quick Disabilities of the Arm, Shoulder and Hand; RoM, range of motion subscale; s, score; SD, standard deviation; SST, simple shoulder test; VAS, Visual Analog Scale.

When comparing the initial evaluations and 1 year after surgery (Table 4), it was found that both groups increased their functionality in a statistically significant way (*p* < .001), according to the Constant and ASES score, with greater differences in the RCR+augmentation with LHB group.

**Table 4:** Differences between the initial and final evaluation in the two experimental groups.

	Control			RCR + SCR		
	MD	SD	*p* Value	MD	SD	*p* Value
Constant (s)						
Pain	−8.95	4.22	**<.001**	−9.59	3.44	**<.001**
Activity	−8.69	3.66	**<.001**	−9.53	3.23	**<.001**
RoM	2.05	5.17	.397	−2.95	5.85	.280
Strength	−4.82	5.05	**.016**	−7.70	6.68	**<.001**
Total	−20.80	11.32	**<.001**	−29.77	10.85	**<.001**
ASES (s)						
Subtotal VD	−7.77	6.07	**<.001**	−10.71	4.67	**<.001**
Total	−43.97	18.57	**<.001**	−53.51	13.83	**<.001**

*Note*: Bold values indicate statistically significant at *p* < .05.

Abbreviations: ASES, American Shoulder and Elbow Surgeons; MD, mean of the differences; RoM, range of motion subscale; s, score; SD, standard deviation of the differences.

The MCID for functional outcome was calculated as 4.38. Patients in the RCR+augmentation with LHB group had 24% more patients who reached the MCID compared to the control group (*p* = .03).

When the between‐group comparison was performed excluding the Goutallier categories 3 and 4 (Table 5), since all of these patients presented retear, the Sugaya scale remained statistically different, with more integrity for the RCR+augmentation with LHB group (*p* = .046). This group also showed more functionality using the SST scale (*p* = .029), with the same trend in the Constant total score (*p *= .053), where the higher improvement was achieved in the range of motion (*p* = .002). The retear proportion was slightly lower in the RCR+augmentation with LHB group as well (*p* = .056).

**Table 5:** Healing and functionality of patients in Goutallier grades 1, 2 and 3 according to the experimental group, 1 year after surgery.

	Control (*n* = 34)	RCR + SCR (*n* = 36)	*p* Value
	*M* ± SD	*M* ± SD	
SUGAYA	3.03 ± 1.19	2.5 ± 1.03	.046
Retear (*n*)	10 (29.4%)	4 (11.1%)	.056^a^
Constant classification(s)			
Pain	12.9 ± 3.24	13.35 ± 2.59	.901
Activity	17.91 ± 3.5	18.22 ± 2.51	.950
Range of motion	32.41 ± 7.19	37.11 ± 2.91	.002
Strength	12.28 ± 7.77	14,84 ± 7.58	.155
Total	75.5 ± 17.05	83.49 ± 10.78	.053
ASES (s)			
Subtotal DL	22.62 ± 6.45	25.11 ± 4.76	.116
Total	79.75 ± 18.17	87.55 ± 13.08	.086
SST (s)	72.13 ± 26.86	85.42 ± 18.73	.029
DASH (s)	18.85 ± 18.4	12.06 ± 16.32	.084

Abbreviations: ASES, American Shoulder and Elbow Surgeons; DASH, Disabilities of the Arm, Shoulder and Hand; *M*, mean; s, score; SD, standard deviation; SST, simple shoulder test.

^a^ Chi‐squared test.

### Factors involved in cuff healing

Among the factors explored for cuff healing considering all the patients, the logistic regression model showed a significant influence of the procedure RCR+augmentation with LHB, patients with this procedure had five times more probability of healing in comparison to the RCR with tenotomy group. In the same way, higher initial functionality, considering Constant total scale, also increased healing probability. In the other hand, longer cuffs had less healing probability. The overall model explained 25% of variability in the sample, with no effect of the age of the patients.

## DISCUSSION

The main finding of this study is that RCR using the LHBT for augmentation achieves higher functionality and lower retear rates compared to RCR with tenotomy at a 1‐year follow‐up (Table 3). These differences remained significant even when controlling for factors such as cuff length and initial functionality (Table 5). Additionally, excluding patients with high fatty degeneration still resulted in a lower retear proportion and better functionality in the LHBT augmentation group.

Cuff re‐rupture rates after suture can range from 11% to 94% [22, 24]. Studies indicate that tendons remaining intact after repair lead to better clinical outcomes, whereas retears reduce functionality over time [9, 18, 32, 40]. This study modified a technique initially for massive irreparable cuff tears, using LHBT augmentation to treat reparable cuff tears, which is reproducible, cost‐effective and minimizes postoperative side effects.

In this study, a technique originally for treating massive irreparable cuff tears was modified using the LHBT to treat reparable cuff tears. This LHB augmentation is reproducible, cost‐effective and reduces postoperative side effects. Inspired by Mihata's procedure [25] for SCR with various grafts, this study used LHB to reinforce cuff repair [4, 5, 6]. All patients had reparable cuff tears, and LHB was used to augment tendon repair, effectively reconstructing the superior capsule of the shoulder in its anatomical position, just below the rotator cuff.

Both study groups were homogeneous in clinical and demographic characteristics (Table 1), and both improved functionality post‐surgery (Table 4). However, the LHBT augmentation group showed a significantly lower retear rate. Typically, patients receive double‐row RCR, but this study showed that adding LHBT augmentation drastically increases healing chances, even when considering factors like cuff length, initial functionality, and age.

These results are not applicable to patients with massive or irreparable cuff tears, where LHBT is used as an isolated SCR or in partial cuff repairs [4, 5]. Previous studies also show that LHBT augmentation yields better long‐term clinical results and healing rates compared to other techniques.

In 2020, Boutsiadis et al. [5] published a study comparing three surgical techniques for the treatment of massive posterosuperior RCR: double‐row technique, transosseous‐equivalent technique with a absorbable patch reinforcement, and RCR with augmentation with LHB. In this study, none of the repaired tendons in the augmentation with LHB group showed re‐ruptures, while 25% and 23.3% of the patch and double‐row groups showed structural failures. They obtained higher healing rates of the infraspinatus tendon, and better long‐term clinical results in the group in which they used LHB for SCR as reinforcement of the cuff repair.

Thus, LHBT augmentation can be considered an effective interposition autograft, with sufficient vascularization promoting healing. Some concerns exist about increased pain with LHBT use, but studies show no significant differences in range of motion or postoperative pain compared to other techniques. Furthermore, biomechanical studies indicate that SCR with LHBT is feasible and potentially stronger than alternatives.

Kim et al. [18]. demonstrated satisfactory results using LHBT to reconstruct the anterior cable in large, retracted cuff tears, with comparable outcomes to human dermal patch reinforcement. Re‐rupture rates were 18.2% for LHBT and 8.1% for the dermis patch, suggesting LHBT is a viable reinforcement option.

Some authors consider that LHB would increase pain in the patients, and that treatment through biceps tenotomy in the context of a cuff tear improves functionality and reduces the risk of postoperative pain after repair [37]. Theoretically, the use of the LHB as an autograft for augmentation of a RCR could cause postoperative pain. In the present procedure, the LHB was used in a distal tenotomy, while using the proximal portion for an augmentation of RCR. In the studies in which it has been used, there were no differences in terms of range of motion or postoperative pain between the techniques, suggesting that the proximal part of the biceps does not increase pain once it is disconnected from the distal portion and may be safely used as a local tissue autograft. Furthermore, a cadaveric biomechanical study demonstrates that SCR with LHB is a feasible procedure and is biomechanically equivalent or even stronger than augmentation with LHBT with a fascia lata autograft [12].

Both groups in this study showed significant functional improvement post‐surgery, confirming that tension‐free double‐row RCR, with or without LHB augmentation, is effective [9, 13, 15, 17, 21, 26, 34]. Even patients with retears improved compared to pre‐surgery measurements. Patients with high fatty degeneration showed poor healing outcomes regardless of the surgical technique. None of the patients with Goutallier grades 3 and 4 had successful cuff healing (Figure 7), consistent with previous findings that fatty degeneration is a poor prognostic factor [14, 24, 36]. This highlights the importance of early intervention before irreversible muscle degeneration occurs.

### Clinical relevance

This study highlights that RCR with LHBT augmentation significantly improves functional outcomes and reduces retear rates compared to traditional methods. This technique offers a cost‐effective and reproducible method to enhance healing, making it a valuable option in rotator cuff repair.

### Beneficiaries and ideal candidates

Patients with reparable rotator cuff tears, especially those without high levels of fatty degeneration, benefit most from this technique. Ideal candidates are those who are younger, have better initial functionality and less extensive cuff damage. Patients with massive or irreparable tears are not suitable candidates for this augmentation method.

#### Limitations of the study

This study has some limitations. The allocation of the patients was sequential instead of randomized, which could contribute to bias in the initial clinical state and functionality registered for each group, although no differences were found between groups in the preoperatory measurements. Another limitation is the reduced number of patients with high fatty degeneration (Goutallier 3 and 4), making it difficult to estimate its influence in the probability of healing since the blank categories made not possible to include this variable as part of the logistic regression model. Finally, the follow‐up for the retear outcome was only of 1 year, it would be valuable to confirm these results after a 2‐year follow‐up period. Furthermore, it should be taken into consideration other clinical aspects such as a partial or complete re‐rupture or the antero‐posterior size.

## CONCLUSION

RCR plus augmentation with LHB tendon achieves a higher healing percentage and a better functional evolution than RCR plus LHB tenotomy 1 year after cuff repair. Fatty degeneration, size of the tear and initial functionality are the main factors involved in cuff healing.

## AUTHOR CONTRIBUTIONS

All authors have contributed to the conduct of the study: they have participated in the surgeries, in the evaluation of the patients and in writing the paper. All authors have contributed to the clinical study and assessment of patients.

## CONFLICT OF INTEREST STATEMENT

The authors declare no conflict of interest.

## ETHICS STATEMENT

The study is part of the doctoral thesis of the main author, which has been reviewed by the ethics committee of the La Fe Hospital in Valencia and supervised by the Catholic University of Valencia San Vicente Mártir. All patients were informed of their participation in the study and signed an informed consent.

## Data Availability

The study data are available in the patient files and medical records at the Manises hospital.
